# Avoiding nasogastric decompression in cases of small bowel obstruction: Truth or dare?^☆^

**DOI:** 10.1016/j.sopen.2022.10.011

**Published:** 2022-11-07

**Authors:** Chad G. Ball

**Affiliations:** Professor of Surgery and Oncology, Hepatobiliary and Pancreatic Surgery, Trauma and Acute Care Surgery, Foothills Medical Center, 1403-29 Street NW, Calgary, Alberta T2N 2T9, Canada

Dr. Livingston and colleagues have published an interesting review of the utility of nasogastric tube (NGT) decompression for patients with small bowel obstruction (SBO). In doing so, they have also highlighted a challenge to a fundamental tenet of gastrointestinal surgery. Chatting about dragons is fun, but slaying them is even better. To that end, the authors have offered us as readers a comprehensive discussion of NGT indications, SBO physiology, and limited clinical outcomes associated with avoidance of NGTs.

The primary fear driving the long-standing traditional use of NGTs remains the possibility of aspiration. When aspiration is volumous (particularly in the elderly, and in patients with poor cardiopulmonary reserve), complications frequently include intubation with mechanical ventilation in the critical care suite, as well as mortality. It has also become clear that many gastrointestinal operations of significant magnitude (colorectal resections, pancreatoduodenectomies, hepatic resections, partial gastrectomies, etc.) do not mandate postoperative NGT use. The strong associated literature supporting this viewpoint has led to the widely employed fast track postoperative pathways we apply to many of our elective/scheduled complex laparotomies. As a result, the last bastion remains the patient who arrives at the hospital with a partial small bowel obstruction in the absence of a planned surgery. Can the same concepts be applied to this scenario?

Functioning NGTs clearly prevent massive life-threatening aspiration (although they may also increase more continuous micro-aspirations). They also serve to decompress the enlarged stomach and therefore limit gastric distention. Gastric distention itself can be a significant concern leading to hemodynamic compromise, intra-abdominal hypertension and/or distracting pain. As a result, there are clearly patients who require NGT decompression in the context of a SBO (complete obstructions, poor physiologic reserve, active vomiting, unstable vital signs, stomachs full of food/material, etc.). The question remains however: is there a subset of patients who can safely avoid NGT decompression? In their analysis of the published literature, 36% of patients did not require NGT placement in the context of an acute SBO. Although that proportion feels inherently reasonable based on high volume experience, the implications of this collective observation remain extremely limited and difficult to extrapolate across centers. More specifically, these studies are small and retrospective descriptions, as opposed to randomized controlled trials. The guaranteed impact of significant selection and publication bias is too overwhelming to ignore. At the end of the day, this is a question that can only be truly answered by prospective randomized methodology.

Experience would anecdotally suggest that cognitively intact patients with at least moderate physiologic reserve, and a partial SBO of limited duration (perhaps with a repeated history of the same issue) may be reasonable candidates to avoid NGT decompression. Akin to injured patients who are admitted for observation following anterior abdominal stab wounds however, these patients with SBO must be frequently reassessed and adequately monitored for potential failure and/or complications.

In summary, Livingston and colleagues have established there is clearly a subset of patients with SBO who can be safely managed without formal NGT decompression. This concept rails against tradition and remains controversial within some global destinations (more than others). The challenge for this publication group, and others to follow, is to provide working surgeons with the tools to identify these patients through the completion of randomized trials.

